# Dr. Hall’s Medicines

**Published:** 1877-04

**Authors:** 


Dr. Hall's Medicines.—Some of the favorite remedies employed by the late Dr. Hall are prepared and sold by Hall & Ruckel, Wholesale Druggists and Importers, at 218 Greenwich Street, New York. Dr. Hall's Spirometer is also sold by them. See their advertisement in this Journal.

